# Functions of Rab Proteins at Presynaptic Sites

**DOI:** 10.3390/cells5010007

**Published:** 2016-02-06

**Authors:** Beyenech Binotti, Reinhard Jahn, John Jia En Chua

**Affiliations:** 1 Department of Neurobiology, Max-Planck-Institute for Biophysical Chemistry, Göttingen 37077, Germany; 2 Interactomics and Intracellular Trafficking laboratory, Department of Physiology, Yong Loo Lin School of Medicine, National University of Singapore, Singapore 117456, Singapore; 3 Neurobiology/Ageing Programme, National University of Singapore, Singapore 117456, Singapore; 4 Research Group Protein trafficking in synaptic development and function, Department of Neurobiology, Max-Planck-Institute for Biophysical Chemistry, Göttingen 37077, Germany

**Keywords:** Presynapse, synaptic transmission, small GTPases, Rabs, intracellular membrane trafficking, synaptic vesicle cycle, autophagy

## Abstract

Presynaptic neurotransmitter release is dominated by the synaptic vesicle (SV) cycle and entails the biogenesis, fusion, recycling, reformation or turnover of synaptic vesicles—a process involving bulk movement of membrane and proteins. As key mediators of membrane trafficking, small GTPases from the Rab family of proteins play critical roles in this process by acting as molecular switches that dynamically interact with and regulate the functions of different sets of macromolecular complexes involved in each stage of the cycle. Importantly, mutations affecting Rabs, and their regulators or effectors have now been identified that are implicated in severe neurological and neurodevelopmental disorders. Here, we summarize the roles and functions of presynaptic Rabs and discuss their involvement in the regulation of presynaptic function.

## 1. Introduction

Rab proteins comprise the largest subgroup of the Ras family of small GTPases [1]. They are evolutionarily conserved and occur in all eukaryotes. Since their initial discovery in yeast and mammals [2,3], our knowledge about their function and molecular mechanisms of action has grown exponentially. Rab proteins are capable of alternating between a GTP bound and a GDP bound state corresponding to the active and inactive states, respectively. An assortment of GTPase-activating proteins (GAP) and guanine nucleotide exchange factors (GEF) act to regulate GTP hydrolysis or the exchange of GDP with GTP [4,5,6]. In addition, nucleotide cycling of Rab proteins is tightly coupled to their membrane association/dissociation cycle, a process requiring two additional factors, Rab GDP dissociation inhibitor (Rab GDI) and GDI-displacement factor (GDF) [7]. Thus, the switching between the active and inactive state is subjected to an exquisite level of spatial and temporal coordination.

Rab proteins exert their function by recruiting specific effectors from the cytoplasm exclusively to the GTP-state of the protein. GTP binding induces major conformational changes in a specific region called switch I and switch II that are exposed on the surface of the Rab proteins to which effectors bind [8]. Although structurally similar, variations in the interface of switch regions confer unique specificity in the interaction of individual Rabs with their respective effectors, thereby creating the impressive diversity in Rab protein function and localization [9].

Rab proteins are essential regulators of various intracellular membrane trafficking steps in the secretory pathway such as cargo selection during vesicle formation, vesicle transport, tethering, and docking. Indeed, Rab proteins are crucial for defining the identity of subcellular membranes, thus serving as molecular zip codes in membrane traffic [10,11]. Each subcellular compartment or trafficking organelle has its own set of Rab proteins controlling its biogenesis, maturation, and transition as well as interaction with other membranous compartments. This includes not only trafficking steps conserved in all eukaryotes but also specialized adaptations such as regulated exocytosis in neurons that govern synaptic transmission. In this mini review, we will focus on presynaptic Rab proteins and highlight their roles in synaptic organization, synaptic transmission, and protein turnover.

## 2. An Overview of Membrane Traffic in Presynaptic Nerve Terminals

**Figure 1:**
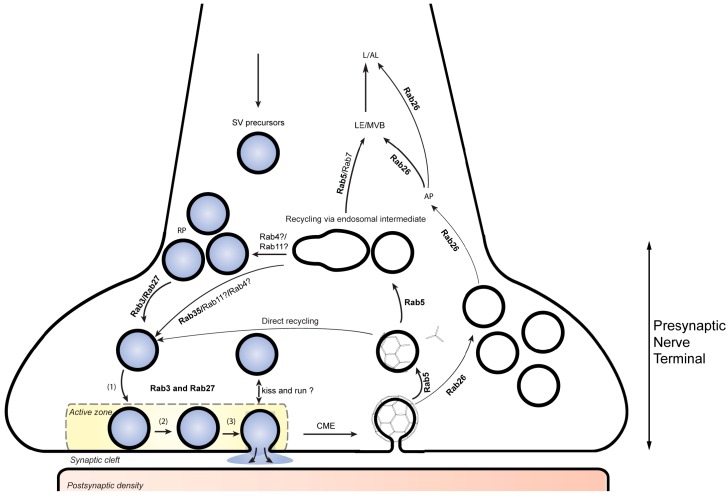
Rab proteins involved in presynaptic function. RAB3 and RAB27B participate in synaptic vesicle (SV) exocytosis while Rab27b has an addition role in synaptic vesicle recycling at the presynaptic nerve terminal. RAB5 participates in the biogenesis and retrieval of synaptic vesicle components through clathrin-mediate endocytosis. Together with RAB5, RAB35 is also thought to be involved in directing these components through early endosomal compartments that contribute to the reformation of SVs replenishing the SV pool. RAB26 is associated with a subset of recycled SVs and directs them for autophagic degradation. SV, synaptic vesicle; RP, reserve pool (1), docking; (2) priming; (3), fusion. CME, clathrin mediated endocytosis. AP, autophagy; LE/MVB, late endosome/multivesiclular body; L/AL, lysosome/autolysosome.

Synaptic transmission is mediated by the regulated release of neurotransmitters from presynaptic nerve terminals (Figure 1). Before release, neurotransmitters are stored in small trafficking organelles, termed synaptic vesicles, some of which are docked to specialized regions of the presynaptic plasma membrane known as active zones [12,13]. Upon arrival of an action potential, Ca^2+^ ions enter the nerve terminal and trigger exocytosis of synaptic vesicles. After exocytosis, the SV membrane is retrieved by endocytosis and SV are locally regenerated to allow for another round of exo-endocytotic membrane cycling [14].

The molecular machineries involved in both Ca^2+^-dependent exocytosis and endocytosis were intensely studied during the past decades, and most of the molecular components are known. Evidently, the key steps in synaptic vesicle cycling are derived from conserved trafficking steps common to all eukaryotes but contain specialized adaptations. Like most eukaryotic fusion events, exocytotic membrane fusion is mediated by SNARE proteins and requires Sec1/Munc18-like (SM) proteins and the complexes associated with tethering containing helical rods (CATCHR) proteins while Ca^2+^ regulation is conveyed by the specialist proteins synaptotagmin and complexin [15]. Similarly, endocytosis is dominated by clathrin-mediated endocytosis (CME) that also involves conserved components such as the clathrin components and adaptor complexes, in addition to synapse-specific components such as AP180 and epsin [16]. Along with CME, a fast and clathrin-independent endocytotic pathway operates in the synapse but whose molecular foundation is less clear [17,18]

A unique feature of nerve terminals is the capacity to locally regenerate synaptic vesicles from endocytosed membranes without the involvement of central trafficking platforms such as the Golgi complex. However, the pathways leading to vesicle re-formation are still debated. Synaptic vesicles may be regenerated directly from endocytosed membrane vesicles without an additional trafficking step. For instance, following clathrin uncoating, the resulting vesicles may directly be loaded with neurotransmitter and enter the pool of synaptic vesicles. Alternatively, endocytosed vesicles may need to pass through an early endosomal compartment from which SVs re-form by budding [19]. Also, only limited information is available about the biosynthetic and degradative pathways that are responsible for the creation of new SV and the elimination of old SV or damaged SV components. SV precursors are derived from the *trans-*Golgi Network and are transported through the axon to the nerve terminal, but it is unclear whether they are fully functional upon reaching synapses or need to undergo fusion-fission events before becoming competent for regulated exocytosis [20,21]. Similarly, the degradation of SVs or SV components is likely to involve proteasomal, lysosomal, and autophagosomal pathways but their relative contribution is not understood.

As all eukaryotic trafficking steps, the synaptic vesicle cycle is governed by specific Rab GTPases. These include sets of specific Rabs involved in exocytosis (RAB3 family), in endosomal function (RAB5, and probably also RAB4, RAB11, and RAB35) and, as recently discovered, Rab proteins involved in autophagosomal function (RAB26 and RAB33).

## 3. Synaptic Vesicle Docking and Exocytosis by Rab3-Related Proteins

The members of the RAB3 protein subfamily represent the most abundant small GTPases in the brain. They include four closely related homologs (RAB3A, RAB3B, RAB3C, and RAB3D) of which RAB3A, RAB3B, and RAB3C are highly expressed in neurons where they specifically localize to synaptic vesicles [14,22]. RAB3A is best investigated and was shown to be involved in regulating the efficiency of neurotransmitter release in neurons [23,24]. RAB3-GTP is bound to the membrane of SVs from which it dissociates during neurotransmitter release, concomitant with GTP hydrolysis [25,26]. However, though mice deficient in all RAB3 isoforms die immediately after birth, only a surprisingly mild reduction (30%) in neurotransmitter release was observed [27]. This may be due to functional redundancy with the structurally related RAB27B which is also expressed at high levels on the membrane of synaptic vesicles, partially overlapping with RAB3 and which shares with RAB3 a common set of regulators and effectors [28,29,30]. Indeed, overexpression of RAB27B mutants (the GTP and GDP preferring variants) caused a strong reduction in SV recycling [22]. Furthermore, impairment of synaptic function was observed upon knockout of RAB3GEF in mice and in *Caenorhabiditis elegans* [31,32,33]. RAB3GEF regulates GTP-exchange of both RAB3 proteins and RAB27B [34]. However, it remains to be established whether RAB27B is a true functional isoform of RAB3 or whether it performs additional functions [35,36]

How are the RAB3 family members regulating exocytosis? Two effectors are known: rabphilin and RIM [28,29,37]. Whereas knockout of rabphilin had only minimal effects on neurotransmitter release [38], knockout of RIM impaired neurotransmitter release although it did not result in a complete inhibition [39,40]. RIM1 is one of the core components of the active zone, which forms a trimeric complex with RAB3 and the CATCHR protein MUNC13 [41,42]. In agreement with the canonical function of Rab proteins in tethering vesicles to target membranes, RIM1 and MUNC13 were proposed to recruit SVs to the active zone and ready them for release [42,43]. Unlike in other trafficking steps, however, recruitment of SVs to the active zone is not only dependent on Rabs, but also on several other factors including the SNAREs themselves [44]. This may explain why knockout of the exocytotic Rab machinery in neurons does impair but not abrogate docking and fusion as it does in other trafficking steps such as constitutive exocytosis in yeast [45,46].

## 4. GTPases Involved in Recycling of SVs

In addition to these exocytotic Rabs, careful analyses of the vesicle composition also confirmed the presence of a specific subset of endosomal Rabs on highly purified synaptic vesicles [22]. These include RAB4 and RAB5 (early endosome, EE) as well as RAB4, RAB11, and RAB35 (recycling endosomes, RE) [47,48]. In addition, Rab proteins associated with autophagy were also identified on these vesicles (e.g., RAB26 and RAB33) suggesting their involvement in eliminating old/damaged synaptic vesicle proteins via the autophagy pathway (see the next section).

Recycling of SVs via the clathrin-mediated pathway presumably occurs via an endosomal intermediate [16,19]. However, the origins and identity of this compartment remains a subject of debate. Of the endosomal Rab proteins, RAB5 coordinates trafficking in the early endocytic events by recruiting a host of RAB5-specific effectors to endocytic vesicles targeted for fusion [49,50]. The involvement of RAB5 in SV recycling was uncovered when the GTPase was found to label an endosomal compartment present at nerve terminals [51,52]. Subsequent experiments additionally showed that RAB5 was directly involved in synaptic vesicle recycling and SV morphology, with RAB5 mutants affecting the formation of these compartments and impairing SV recycling [53,54].

In addition to RAB5, RAB35 is another small GTPase that has been more recently shown to participate in the recycling of SVs via the presynaptic endosomal compartment. In *Drosophila*, expression of constitutively active RAB35 or loss of function of its corresponding GAP, Skywalker, promotes retrieval of synaptic vesicles via endosomal intermediates and increases synaptic transmission indicating that RAB35, like RAB5, is important for regeneration of new synaptic vesicles [55].

## 5. Degradation and Turnover of SVs

Regulated turnover of proteins is essential for synaptic plasticity and synaptic remodeling. Periodic turnover of synaptic vesicles and their proteins is also necessary to remove damaged components. While proteasomal degradation of proteinaceous synaptic material is documented, less is known about how synaptic vesicle membranes and their associated proteins are removed [56,57]. Apart from proteasomal degradation of membrane-associated proteins, it is conceivable that membrane constituents are delivered to lysosomes after sorting into multivesicular bodies by the ESCRT pathway. Indeed, multivesicular bodies containing synaptic proteins were found in axons, suggesting that this pathway also operates in nerve terminals [58].

Besides the proteasome pathway, autophagic clearance of cellular components also plays important roles in neurons [59,60]. Autophagosomes can form in distal axons and are transported back to neuronal cell bodies for degradation following fusion with lysosomes [61,62]. Several Rab proteins (such as RAB7, RAB8, and RAB33) and their effectors are known to be involved in regulating various steps in the autophagy pathway [63]. Recently, we discovered a direct link between the turnover of recycled SV and the autophagic pathway that involves RAB26.

In neurons, RAB26 is enriched in SVs and adorns a subset of SVs that have undergone recycling [64]. Overexpression of constitutively active RAB26 causes the appearance of large intracellular vesicular aggregates containing synaptic vesicle proteins such as synaptobrevin and RAB3. Remarkably, these aggregates are positive for autophagosomal markers such as ATG16L1 and LC3B. Strikingly, RAB26 interacts with a complex of ATG16L1-ATG5, indicating that the GTPase plays a direct role in earmarking synaptic vesicles for degradation via the autophagyc pathway.

In addition to RAB26, the two isoforms of RAB33 have also been shown to bind to ATG16L1 [65]. While RAB33A is highly expressed in the brain, Rab33B is ubiquitously present. Interestingly, the interaction of RAB33B with Atg16L1 regulates autophagosome formation. In comparison to this, the interaction of RAB33A with Atg16L1 does not appear to play a direct role in autophagy but serves rather to regulate hormone secretion and mediate anterograde transport of Golgi-derived vesicles involved in the exocytosis in an autophagy-independent manner [66,67]. Since RAB26 and RAB33B both share a common effector, it will be important to determine if RAB26 and RAB33 potentially overlap in their roles in autophagy in neurons to regulate the turnover of SVs.

## 6. Presynaptic Rabs and Their Involvement in the Neuronal-Associated Disorders

Many mutations and physiological abnormalities affecting synaptic Rabs have now been identified that contribute to neurodegenerative and neurodevelopmental disorders. Aggregates of α-synuclein trap RAB3A and prevent its interaction with rabphilin, suggesting that exocytosis and neurotransmitter release are affected in Lewy´s Body diseases [68]. Patients affected by the Warburg-Micro-syndrome and the Martsolf-syndrome, which is a more attenuated form of the former, carry mutations in the catalytic and non-catalytic domains of RAB3GAP. Both are inheritable forms of neurological diseases exhibiting developmental abnormalities and intellectual disability [69,70]. Patients affected by X-linked, non-specific mental retardation harbor mutations in αGDI (Rab GDP-dissociator inhibitor). One such mutation (L92P) affects RAB3A recycling which might indicate that proper SV cycling is fundamental for mental development [71]. Interestingly, loss of αGDI in mice affects short term memory and social behavior and *Rab3a* null mice also exibit impaired learning, albeit with different phenotypes compared to the ones observed for αGDI [72,73]. Recent work also revealed that knock out of *Rab3b* gene affects long term depression (LTD) of inhibitory synapses and enhanced reversal learning in mice [74].

Abnormalities in the endosomal pathway are found in some of the commonly known neurodegeneratve diseases such as Alzheimer’s (AD), Huntington’s (HD), and Parkinson’s disease (PD) (for review see [75]). For instance, RAB5 appears to play a role in the internalization of exogenous α-Synuclein and overexpression of constitutively active RAB5 or its effector Rabaptin leads to the formation of intracellular inclusions in cultured neurons resembling Lewy´s bodies [76]. ALS2/alsin is a RAB5GEF and its depletion is responsible for different recessive forms of motor neuron diseases. This implies that RAB5 activity, and by inference the endosome pathway, plays a crucial role in contributing to severe motor neurons disorders such as Amyotrophic Lateral Sclerosis (ALS) [77,78]. Huntingtin-HAP40, a RAB5 effector, is upregulated in Huntington’s disease (HD) suggesting that RAB5 is also implicated in this disorder [79,80].

Many neurological diseases show defects in recycling endosomes [81]. Recent studies uncovered mutations in RAB35GAP (also known as Skywalker or TBC1D24) in patients with familial infantile myoclonic epilepsy or focal epilepsy with intellectual disability syndrome [82,83]. RAB11 family (including the ubiquitously expressed RAB11A, the neuronal RAB11B and the epithelial RAB25) is also an important member of the recycling pathway and deregulated activity of RAB11 is found in many neuronal disorders [11,84,85]. For instance, the function of RAB11 was found to be inhibited in many models of HD [86]. RAB11 is also implicated in Alzheimer’s disease by its ability to bind presenilin 1 and presenilin 2, which are components of the γ-secretease responsible for the generation of Aβ peptides [87].

Intracellular membrane trafficking pathologies might be causative or a consequence of many neurodegenerative diseases. Studying the role of the small GTPases and their effectors and regulators at the molecular level would improve our understanding the pathology of these human diseases and may in turn offer new avenues for therapeutic intervention.
